# Reflex‐based grasping, skilled forelimb reaching, and electrodiagnostic evaluation for comprehensive analysis of functional recovery—The 7‐mm rat median nerve gap repair model revisited

**DOI:** 10.1002/brb3.813

**Published:** 2017-09-06

**Authors:** Maria Stößel, Lena Rehra, Kirsten Haastert‐Talini

**Affiliations:** ^1^ Institute of Neuroanatomy and Cell Biology Hannover Medical School Hannover Germany; ^2^ Center for Systems Neuroscience (ZSN) Hannover Hannover Germany

**Keywords:** electrophysiology, grasping test, rat median nerve, staircase test

## Abstract

**Introduction:**

The rat median nerve injury and repair model gets increasingly important for research on novel bioartificial nerve grafts. It allows follow‐up evaluation of the recovery of the forepaw functional ability with several sensitive techniques. The reflex‐based grasping test, the skilled forelimb reaching staircase test, as well as electrodiagnostic recordings have been described useful in this context. Currently, no standard values exist, however, for comparison or comprehensive correlation of results obtained in each of the three methods after nerve gap repair in adult rats.

**Methods:**

Here, we bilaterally reconstructed 7‐mm median nerve gaps with autologous nerve grafts (ANG) or autologous muscle‐in‐vein grafts (MVG), respectively. During 8 and 12 weeks of observation, functional recovery of each paw was separately monitored using the grasping test (weekly), the staircase test, and noninvasive electrophysiological recordings from the thenar muscles (both every 4 weeks). Evaluation was completed by histomorphometrical analyses at 8 and 12 weeks postsurgery.

**Results:**

The comprehensive evaluation detected a significant difference in the recovery of forepaw functional motor ability between the ANG and MVG groups. The correlation between the different functional tests evaluated precisely displayed the recovery of distinct levels of forepaw functional ability over time.

**Conclusion:**

Thus, this multimodal evaluation model represents a valuable preclinical model for peripheral nerve reconstruction approaches.

## 1 INTRODUCTION

Peripheral nerve injuries (PNIs) are a major cause of morbidity, especially as in most cases the upper limbs are affected (Deumens et al., 2010; Kouyoumdjian, 2006). In 10.2% of all hand injuries, a peripheral nerve reconstruction is required, whereas in two third of the cases, the digital nerves are damaged (Renner, Cserkuti, & Hankiss, 2004). Autologous nerve grafts (ANGs) remain the gold standard for nerve reconstruction. Usage of ANGs, however, leads to considerable donor side morbidity. Therefore, autologous muscle‐in‐vein grafts (MVGs), leading only to minor donor side morbidity (Manoli, Schulz, Stahl, Jaminet, & Schaller, 2014), represent promising alternative digital nerve grafts (Manoli, Schulz, et al., 2014; Marcoccio & Vigasio, 2010; Tos, Battiston, Ciclamini, Geuna, & Artiaco, 2012) as do several clinically approved biodegradable nerve guidance conduits (NGCs) (Bertleff, Meek, & Nicolai, 2005; Donoghoe, Rosson, & Dellon, 2007; Lohmeyer, Siemers, Machens, & Mailander, 2009; Meyer et al., 2016).

To study novel approaches in peripheral nerve reconstruction and nerve graft tissue engineering, the sciatic nerve (SN) is easily accessible and its length provides the opportunity of addressing long gap repair (Tos et al., 2009) with a number of functional tests (Navarro, 2016). The SN model, however, is often inflicted by automutilation behavior and the development of hamstring contractions, eventually reducing the number of animals available for analysis and the significance of the results (Navarro, 2016; Weber, Proctor, Warner, & Verheyden, 1993).

The rat median nerve (MN) model is not compromised by automutilation behavior or secondary changes in the musculoskeletal system and deserves to our opinion a revisit. Although first described already 22 years ago (Bertelli & Mira, 1995), until today no reports exist on comprehensive evaluation of the different motor functions transmitted via the MN. Several functional evaluation techniques have been described in mostly independent use in literature (Bertelli & Mira, 1995; Galtrey & Fawcett, 2007; Montoya, Campbell‐Hope, Pemberton, & Dunnett, 1991; Wang, Spinner, Sorenson, & Windebank, 2008).

The aim of this study was to advance the commonly known rat MN injury and repair model by defining a comprehensive functional readout. We anticipated that the advanced model would qualify for the assessment of different nerve reconstruction approaches with regard to the onset, progress, and completeness of motor recovery. We also intended to reduce the number of animals used. While in the rat SN model it is ethically not acceptable to do a bilateral injury and repair, the relatively mild general impairment induced by a bilateral MN injury is less problematic.

For bilateral bridging of 7‐mm rat MN gaps, ANGs and MVGs were used as model grafts. The defect lengths of 7 mm matched 70%–80% of the length of the humerus along which the MN travels on its trajectory from the axilla toward the elbow. For revisiting and advancing the rat MN model, we first refined the commonly applied grasping test by developing a video‐based scoring system that allowed the categorization of individual paw usage abilities following bilateral reconstruction. Secondly, the staircase test was included to reflect the accuracy of motor reinnervation. The test is commonly applied to examine alterations in fine motor skills following brain injuries or in the course of neurodegenerative diseases of the central nervous system (Effenberg et al., 2014; Klein, Sacrey, Dunnett, Whishaw, & Nikkhah, 2011; Montoya et al., 1991), but was rarely described in its use to study recovery from PNIs (Galtrey & Fawcett, 2007; Pagnussat Ade, Michaelsen, Achaval, & Netto, 2009). Thirdly, we applied serial electrodiagnostic recordings of evoked compound muscle action potentials (CMAPs), also commonly applied in the rat SN model (Korte, Schenk, Grothe, Tipold, & Haastert‐Talini, 2011; Navarro, 2016). We evaluated the reinnervation of the thenar muscle, which is together with all the finger flexors solely innervated by the MN (Bertelli & Mira, 1995). So far, only one report exists on serial evaluation of thenar muscle reinnervation (Wang, Sorenson, Spinner, & Windebank, 2008), while others report invasive endpoint recordings from the finger flexors (Sinis et al., 2005; Werdin et al., 2009).

## 2 MATERIALS AND METHODS

### 2.1 Animals and surgical procedure

Animal experiments were approved by the animal care committee of Lower‐Saxony, Germany (approval code: 33.12‐42502‐04‐15/1761). Sixteen adult female Lewis rats (body weight at the day of surgery: 185.3 ± 0.8 g) were housed in groups of four under standardized housing conditions (+22.2°C; humidity 55.5%; light/dark cycle 14 hr/10 hr) with food and water provided ad libitum. During staircase test phases, food was restricted to 12 g per animal and day and body weight was controlled every other day (tolerable weight loss up to 15%). The mean body weight of the animals did, however, still increase continuously over time until 12 weeks postsurgery. At least 72 hr were kept between the end of restrictive feeding (for staircase test) and anesthesia.

Prior to surgery, functional healthy state of all animals was assessed by means of grasping test, staircase test, and noninvasive electrophysiological measurements. All surgeries and noninvasive electrophysiological measurements were performed under aseptic conditions. Deep anesthesia was administered by intraperitoneal injection of chloral hydrate (370 mg/kg, Sigma‐Aldrich Chemie GmbH, Germany). For sufficient analgesia during surgery and the 2 days after as well as during electrodiagnostic evaluation, butorphanol (0.5 mg/kg, Torbugesic^®^; Pfizer GmbH, Germany) was injected subcutaneously. Prior to nerve transection, bupivacaine (0.25%, Carbostesin^®^; AstraZeneca GmbH, Germany) and lidocaine (2%, Xylocain^®^; AstraZeneca GmbH, Germany) were additionally applied locally on the exposed nerve.

In the MVG group animals, the two respective grafts were assembled first by bilaterally exposing the superficial epigastric vein through a 1‐cm incision in the inguinal region. Following cauterization, a 7‐mm‐long vein segment was removed and rinsed in a vasodilatory heparin‐mepivacain solution (0.5% of Heparin‐Natrium‐25000‐ratiopharm^®^, Ratiopharm GmbH, Germany, and 0.5% of Scandicain^®^ 2%, AstraZeneca GmbH, Germany, in NaCl 0.9%, B. Braun Melsungen GmbH, Germany). Then, some muscle fibers of adapted length were separated from the gracilis muscle and longitudinally introduced into the purged vein.

For nerve surgery, the MN was approached through a 1‐cm incision parallel to the humerus in the axillary region. Distal transection was first uniformly performed proximal to the point where the brachial artery crosses the MN and followed by the second transection 7‐mm proximal to this point. In case of MVGs sutured between the nerve ends, the nerve piece was removed and further processed for either immunohistochemistry or nerve morphometry to serve as a healthy nerve control. In case of ANGs, the nerve piece was reversed and rotated 180° before suturing it between the nerve ends. This procedure results in vessels and axonal tracts that no longer exactly fit between the nerve transplant and the sutured nerve ends; thus, matching the situation after clinical nerve grafting. At each nerve end, two epineural 9‐0 stitches were applied for suturing the grafts.

### 2.2 Grasping test

**Figure 1:**
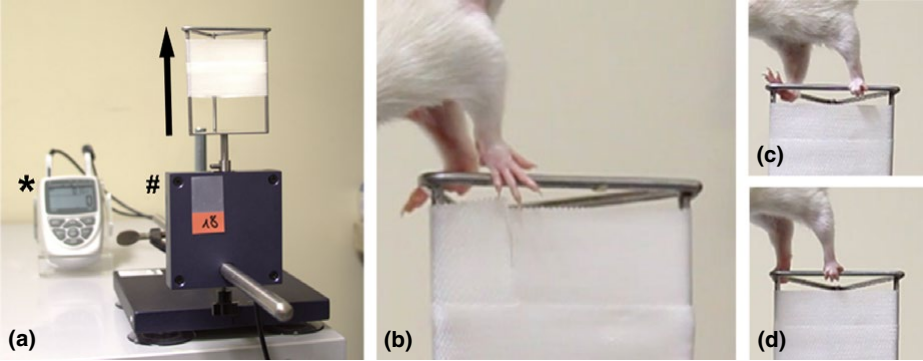
Photograph of the grasping test device and picture details taken from video sequences as an example of the grasping ability categorization. (a) The grasping device was mainly composed of the digital force transducer (indicated by #) that was attached on a heavy base plate and cable connected to a Grip Strength Test Meter (indicated by *) for measurement of the forelimb grip force in [g] on the one side while on the other side the enhanced grasping frame was screwed. (b–d) The animal was held by its tail and was gently passed over the enhanced grasping frame until it touched the upper metal bar (b). Due to the grasping reflex, the animal immediately grasps the bar in case of proper innervation of the finger flexors (d). Then, the animal was slowly lifted while it was pulling the grasping frame in the indicated direction (arrow in a). In the first weeks following surgery, animals were not able to grasp the metal bar after touching it (b). After a while, reinnervation led to regained finger flexion even though in the beginning, this usually did not occur at the same time in both paws (c). That is why video analysis is an essential tool to evaluate bilaterally treated animals. The white tape seen in all pictures was utilized to avoid holding the bar via wrist flexion

Reflex‐based movement (Tupper & Wallace, 1980) during the recovery of finger flexion progressing to grip force recovery was studied weekly from the second week postreconstruction onward. A Grip Strength Test Meter (IITC Life Science Inc., USA) with an enhanced grasping frame (Papalia, Tos, Stagno d'Alcontres, Battiston, & Geuna, 2003) was used (Figure 1a). A Canon EOS‐1D Mark IV camera (Canon Deutschland GmbH, Germany) was mounted on a tripod to record the grasping abilities of each single paw of individual animals (Figure 1b–d). The grasping behavior was categorized as follows: category 1—no ability for finger flexion while touching the grasping bar (Figure 1b); category 2—ability to encompass the grasping bar (closing digits around the bar) but disability to hold on to it while being slowly lifted (Figure 1c,d); category 3—ability to grasp and pull the bar with a recordable force (gross motor skills). The animals were presurgically habituated to be held upside down for a few seconds while simultaneously recording a healthy baseline reference value.

### 2.3 Staircase test

Before surgery, animals were habituated to the experimental procedure in a 3‐week handling period (Galtrey & Fawcett, 2007) including daily training on the final 10 consecutive days. Within the staircase apparatuses, the rats could not turn around while sitting on the central plinth and reaching to the left/right stairs only with the respective paw (Klein & Dunnett, 2012). Both stairs are composed of seven steps, each equipped with three sugar pellets (AIN‐76A Rodent Tablet 45 mg IRR, Lot number: 12SEP17RTD1, TestDiet™, USA). Remaining sugar pellets on the stairs were counted after 15 min. After 7 days of training, a performance plateau was reached by 15 animals which substantially improved over time, while one animal completely refused participation in the test (excluded from further evaluation). Healthy baseline reference values for each individual paw were calculated as mean maximum number of pellets retrieved in the last 3 days of training (serving as control values for the same animal/paw).

Retesting was performed at 4, 8, and 12 weeks postsurgery. Restrictive feeding and rehabituation were induced 7 days before each test cycle, the test was performed daily for 7 consecutive days, and the mean performance of the last 3 days was calculated. To exclude the impact of individual paw preferences, postsurgical achievements were evaluated as a percentage of the individual healthy baseline scores.

### 2.4 Noninvasive electrophysiological recordings

**Figure 2:**
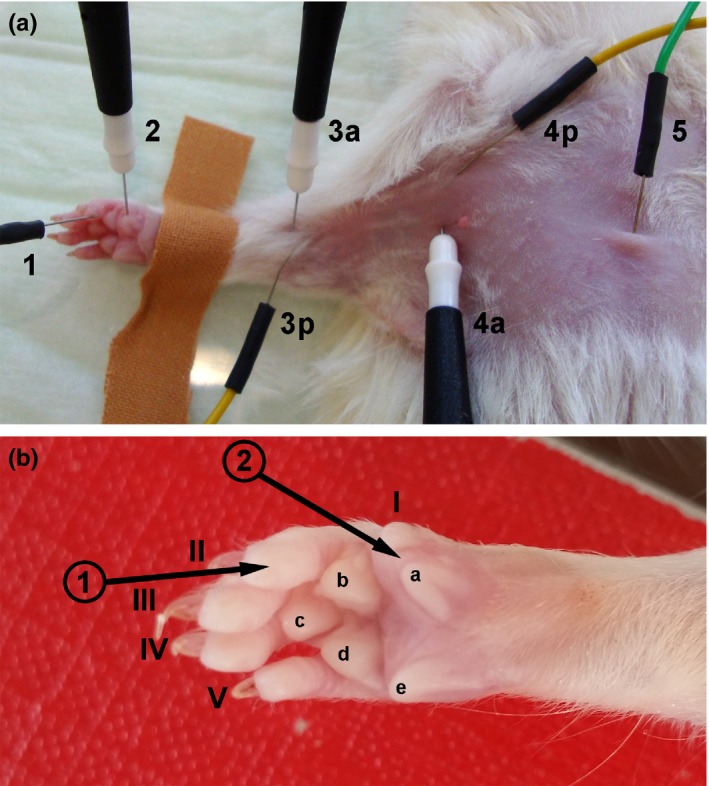
Photographs demonstrating the ventral view of electrode positioning during noninvasive electrophysiological recordings and of the magnified palmar view of the right paw to clarify exact positioning of the recording electrode. (a) Proximal stimulation was achieved by introducing active (4a) and passive (4p) stimulation electrodes close together in the axillary region while distal stimulation was performed by placing active (3a) and passive (3p) stimulation electrodes close together at the elbow. The ground electrode (5) was subcutaneously introduced above the sternum. (b) As indicated by the black arrows, the recording electrode (2) was positioned at the thenar muscle between the rudimentary thumb (I) and the medial walking pad (a), while the reference electrode (1) was placed in the finger tip of the second digit (II)

Noninvasive electrophysiological recordings were performed once presurgery and every 4 weeks postsurgery in anesthetized animals placed in supine position (body temperature controlled). Single electrical impulses (100 μs, 1 Hz) were delivered by transcutaneous monopolar needle electrodes, while stimulus intensity was gradually increased up to 30% supramaximal level. The reconstructed MN was either stimulated proximal to the injury site in the axillary region or distal to the graft at the elbow (Figure 2a). CMAPs were recorded transcutaneously from the thenar muscles (Wang, Sorenson, et al., 2008) (Figure 2a,b). The anatomical situation and the trajectory of the MN ensure that CMAP recordings from the thenar muscle result from MN stimulation, even if in the axillary region the ulnar nerve might easily be costimulated. The reference electrode was placed in the tip of the second digit (Figure 2a,b). Amplitudes of the evoked CMAPs were semiautomatically evaluated (baseline to negative peak of the M‐wave) using a Dantec^®^ Keypoint^®^ Focus device (Natus Europe GmbH, Germany). If no evoked CMAPs or CMAPs with no clear negative M‐wave peak could be detected, a zero value was included into statistical analysis.

### 2.5 Nerve immunohistochemistry

At 8 and 12 weeks postsurgery, four animals per group were sacrificed (each two animals with best and poorest functional results). Upon harvest, the macroscopic appearance was analyzed before the tissue from mid‐graft level to distal coaptation site was fixed overnight (4% paraformaldehyde in phosphate buffered saline (PBS, Dulbecco, Biochrom GmbH, Germany), +4°C, Sigma‐Aldrich Chemie GmbH, Germany) prior to paraffin embedding. Series of 40 blind‐coded cross‐sections (7 μm) were prepared. Hematoxylin and eosin staining (HE) was applied to selected sections and consecutive sections were double stained for neurofilament (NF200) (Meyer et al., 2016) and myelin basic protein (MBP). Briefly, sections were incubated in blocking solution (3% milk powder (Bio Magermilch Pulver, Heirler Cenovis GmbH, Germany) and 0.5% triton X‐100 (Roche Diagnostics GmbH, Germany) in PBS) prior to incubation with the following antibodies one after the other: (i) primary rabbit α‐NF200 antibody (against phosphorylated NFeH, N4142, 1:500, Sigma‐Aldrich GmbH, Germany, RRID:AB_477272, +4°C overnight), (ii) Alexa 488‐conjugated secondary goat α‐rabbit antibody (A11034, 1:1,000, Invitrogen AG, Germany, 1 hr at room temperature (RT)), (iii) mouse α‐MBP antibody (SMI94, 1:200, Covance Antibody Products, Germany, RRID:AB_510039, +4°C overnight), and (iv) Alexa 555‐conjugated secondary goat α‐mouse antibody (A21422, 1:1,000, Invitrogen AG, 1 hr at RT). All antibodies were diluted in blocking solution and all incubation steps were followed by three times washing in PBS. As part of previous studies with the antibodies used herein, standard positive and negative control procedures with omitting primary or secondary antibodies were performed and confirmed the specificity of all antibodies (not presented). After nuclear counterstaining with DAPI (1:2,000 in PBS, Sigma‐Aldrich GmbH), sections were mounted using Mowiol (Calbiochem GmbH, Germany).

Representative photomicrographs were created in multiple image alignments (MIA) using a BX51 microscope (Olympus GmbH, Germany) expanded with a joystick‐controlled microscope stage (MBF Bioscience, USA) and the Stereo Investigator version 11.07 (MBF Bioscience, USA, RRID:SCR_002526).

### 2.6 Histomorphometry

Nerve segments harvested distal to the grafts and healthy nerve samples collected during MVG reconstruction surgery were fixed in Karnovsky solution, rinsed in sucrose‐sodium cacodylate buffer, and postfixed in 1% osmium tetroxide for 1.5 hr before myelin sheath staining with 1% potassium dichromate and hematoxylin (Korte et al., 2011). Finally, samples were epon embedded and semithin cross‐sections (1 μm) were prepared. Myelin staining was enhanced by toluidine blue staining and sections were mounted using Mowiol.

Two randomly selected sections of each specimen (*n* = 8 per group) were evaluated at light microscopic level (BX50 microscope Olympus GmbH, Germany) expanded with a prior controller (MBF Bioscience, USA). Total cross‐sectional area (20× magnification), total fiber number (100× magnification), and mean fiber density were determined using a two‐dimensional procedure (optical fractionator; grid size: 150 × 150 μm²; counting frame size: 30 × 30 μm²) with the help of Stereo Investigator version 11.04 (MBF Bioscience, USA, RRID:SCR_002526). Only the fiber “tops” were counted to cope with the “edge effect” (Geuna, Tos, Battiston, & Guglielmone, 2000). In four randomly selected photomicrographs (100× magnification) of each section, axon and fiber diameter, myelin thickness, and g‐ratio (G‐ratio plug‐in, RRID:SCR_015580, http://gratio.efil.de/, ImageJ version 1.48, National Institutes of Health, USA, RRID:SCR_003070, 10 axons per picture, 80 examined axons per specimen, 320 considered axons per group and time point) were determined. Axon and fiber diameter were calculated following the assumption that both were circular.

### 2.7 Statistics

Statistical analysis was performed using GraphPad Prism version 6.05 (GraphPad Software, USA , RRID:SCR_002798). All values are presented as mean ± SEM or median ± range as indicated. Healthy state reference values were subjected to Wilcoxon matched‐pairs signed rank test to determine significant differences between right and left paw. The proportion of animals per group that displayed successful participation in either the grasping or the staircase test and that showed an evocable CMAP was calculated as percentages (0–100%) and analyzed with the Chi‐Square test to elucidate differences between the ANG and MVG group. All other results were evaluated using two‐way ANOVA (Analysis of Variance) followed by Sidak's posttest or by Tukey's multiple comparison as indicated. Level of significance: *p* < .05.

## 3 RESULTS

### 3.1 Healthy nerve reference values

In order to reduce animal numbers, a 7‐mm gap was reconstructed within the left and right MNs of all animals. Therefore, the healthy nerve initial values of each animal were recorded presurgically and are displayed in Table 1.

**Table 1:** Healthy nerve mean reference values obtained from the three applied functional tests were recorded presurgically to enable both, a bilateral reconstruction surgery but still the comparison of motor recovery with a healthy state

Parameter	Total (*n* = 16 animals)	Right paw (*n* = 16 paws)	Left paw (*n* = 16 paws)
Grasping test			
Maximum force applied (g)	223.4 ± 9.5		
Staircase test			
Maximum number of pellets retrieved	9.1 ± 0.7	10.7 ± 0.8	7.6 ± 0.7***
Maximum number of steps reached	4.6 ± 0.3	4.9 ± 0.3	4.4 ± 0.3**
Noninvasive electrophysiological measurements			
Amplitude height (mV)	5.85 ± 0.33	5.70 ± 0.37	6.00 ± 0.49
Amplitude area (ms·mV)	3.82 ± 0.34	3.63 ± 0.42	4.01 ± 0.41
Nerve conduction velocity (m/s)	30.85 ± 2.42	32.17 ± 2.18	29.54 ± 3.64

As right and left paw were evaluated separately, values were defined individually for both paws compared to a total mean value. Wilcoxon matched‐pairs signed rank test was applied to examine significant differences between contralateral paws (**p < .01, ***p < .001 between right and left paw). Values are given as mean ± SEM.

Female Lewis rats (15 weeks of age) were able to pull the grasping bar with a force 1.2 times higher than their own body weight. While grasping, they used both forelimbs and closed all digits of both wrists around the bar. In the staircase test, 12 of 16 animals demonstrated a strong right paw preference resulting in significantly better results for the right paw compared to the contralateral side (maximum number of pellets retrieved, maximum number of stair steps reached). Due to smaller body sizes, animals at presurgical state were not able to retrieve pellets from the deepest two steps (numbers 6 and 7) while, in contrast, the sixth step was almost reached and eventually swept by the end of the study (12 weeks postsurgery). Electrodiagnostic measurements displayed no significant differences between left and right paws.

### 3.2 Assessment of functional motor recovery

Three different evaluation methods were applied: The grasping test (recovery of individual grasping behavior [fine motor skills] and regained grip force [gross motor skills]), the staircase test (fine motor skills in reaching and grasping), and noninvasive electrodiagnostic measurements (motor recovery in general). Table 2 summarizes the timeline for different degrees of functional motor recovery as displayed from the comprehensive MN testing.

**Table 2:** Timeline of functional motor recovery resulting from successful participation in the grasping and the staircase test and due to evocable compound muscle action potentials (CMAPs) recorded from the thenar muscle upon transcutaneous stimulation of the reconstructed median nerve

Observation method	Group	4 weeks	8 weeks	12 weeks
		Paws/group (%)	Paws/group (%)	Paws/group (%)
Grasping test^a^	ANG	1/16 (6.3%)	14/16 (87.5%*)	8/8 (100.0%)
	MVG	0/16 (0.0%)	9/16 (56.3%)	5/8 (62.5%)
Staircase test^b^	ANG	7/16 (43.8%*)	15/16 (93.8%)	8/8 (100.0%)
	MVG	1/14 (7.1%)	11/14 (78.6%)	6/8 (75.0%)
Electrophysiological recordings^c^	ANG	12/16 (75.0%)	16/16 (100.0%)	8/8 (100.0%)
	MVG	14/16 (87.5%)	15/16 (93.8%)	8/8 (100.0%)

ANG, autologous nerve graft; MVG, muscle‐in‐vein graft. Values are given both as exact numbers (left or right paws successfully participating per group) or as percentages (%). Earliest signs of functional motor recovery were found with the help of electrophysiological recordings while later on, the staircase test revealed an earlier onset of recovery of fine motor skills. Finally, reflex‐based grasping with a force representing most complete gross motor skills displayed the latest onset. Chi‐Square test was applied to examine significant differences between both groups (*p < .05 between ANG and MVG at the same time point). At 8 weeks postsurgery, the number of animals was reduced by 50% for interim histomorphometrical analysis.

a As successful participation, only those paws were counted that were able to pull the grasping bar with a measurable force.

b As successful participation, only those paws were counted that retrieved more than 3.0 pellets because we found that most animals were able to reach the first step with their tongue.

c As successful outcome, only those paws were counted that revealed evocable CMAPs recorded from the thenar muscle.

Earliest evidence of motor recovery was found in the electrodiagnostic measurements. Already at 4 weeks postsurgery, a minimum of 75% of the reconstructed nerves led to evocable CMAPs, irrespective of the graft type applied. At the same time, participation in the behavioral tests was quite poor. Only one animal of the ANG group was able to pull the grasping bar with one paw, while no animals of the MVG group successfully executed the grasping reflex. In the staircase test, ANG‐reconstructed nerves performed significantly better, although still less than 50% enabled successful participation.

Until 8 weeks postsurgery, both groups strongly improved in all three tests applied (highest amount of recovery detected in electrodiagnostic measurements, lowest success rates detected in the grasping test). The grasping test exclusively displayed significant differences between both groups at this stage.

By the end of the observation period, ANG‐reconstructed nerves led to 100% functional motor recovery displayed in all three tests. In contrast, a clear gradation was found in the MVG group nerves, all displaying evocable CMAPs but only 75% successful participation in the staircase test. Interestingly, one of these recovered paws was not able yet to encompass the grasping bar.

More detailed results obtained from the three different functional evaluations methods applied and the final histomorphometrical evaluations follow.

### 3.3 Grasping test

**Figure 3:**
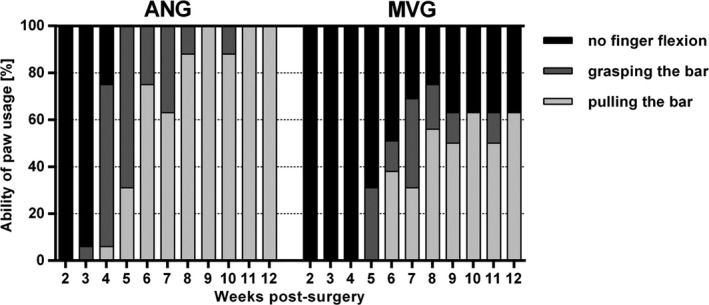
Classification of the individual paw usage abilities based on the weekly performed reflex‐based grasping test revealed the onset and progression of functional motor recovery over 12 weeks postsurgery. With the help of video analysis, an earlier onset of functional recovery was found in autologous nerve graft–reconstructed animals leading to complete functional recovery by the end of observation. Functional recovery of muscle‐in‐vein graft repaired animals was delayed and incomplete at 12 weeks postsurgery (*n* = 16 at 4 and 8 weeks postsurgery; *n* = 8 at 12 weeks postsurgery). No statistical evaluation was applied. Values are given as percentages related to all evaluated paws in the particular group

The individual grasping behavior was classified into three categories as described above (Figure 3). In general, an earlier onset of recovery of gross motor skills was found in ANG‐reconstructed forelimbs leading to 100% recovery at 11 weeks postsurgery. The first animal was able to grab for the bar already at 3 weeks postsurgery while 2 weeks later, all ANG‐reconstructed animals showed at least finger flexion (recovery of reflex‐induced fine motor skills, category 2). From 9 weeks postsurgery onward, all animals additionally applied a certain force to the grasping frame (recovery of gross motor skills, category 3).

In contrast, the functional motor recovery of MVG‐reconstructed forelimbs was delayed and incomplete. First animals were able to grab the frame not before 5 weeks postsurgery. One week later, first animals were able to pull the frame. Until 8 weeks postsurgery, 75% of MVG‐reconstructed forelimbs showed at least finger flexion while 56% also applied a measurable force to the grasping frame.

Following reduction in animal numbers at 8 weeks postsurgery, the outcome almost stabilized within the remaining four animals (eight reconstructed MNs) in both groups. Little up‐ and downturns in the performances during the last 4 weeks (both groups) relate to the fact that animals appeared bored (less motivated to participate) over time by the weekly repeated procedure.

### 3.4 Staircase test

**Figure 4:**
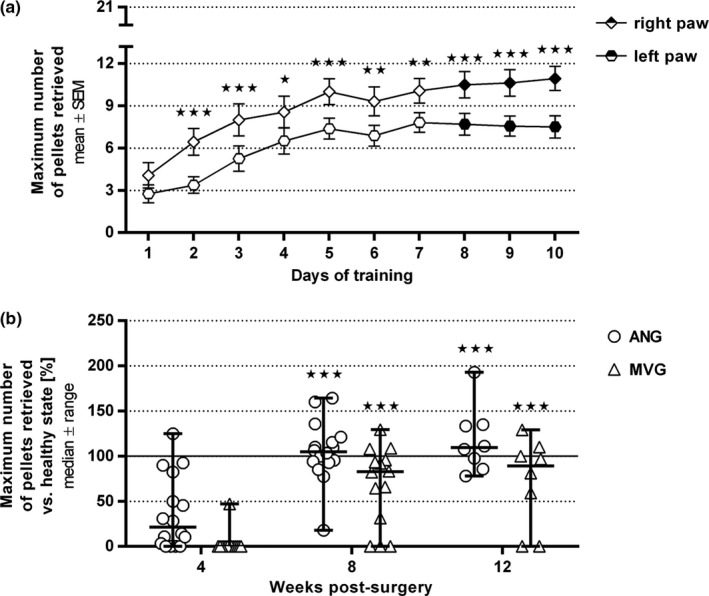
Quantitative results of the staircase test training phase (a) and its following application to evaluate functional motor recovery by means of fine motor skills over 12 weeks postsurgery (b). (a) During 10 consecutive days of training, paw usage abilities constantly improved leading to a plateau phase (from day 7 onward) in each paw by the end of training (*n* = 16 paws per group). The last three achievements (indicated by filled symbols) were taken into account to calculate the healthy state mean reference value for each individual paw. Significant paw preference was found in most animals leading to a better performance with the right paw. Two‐way ANOVA followed by Sidak's multiple comparison was applied to examine significant differences (*p < .05, **p < .01, ***p < .001 vs. contralateral paw). Values are given as mean ± SEM. (b) The staircase test was applied to monitor recovery of motivation‐induced fine motor skills and demonstrated a significant increase in both groups over time without significant differences between the groups (autologous nerve graft [ANG]: *n* = 16 at 4 and 8 weeks postsurgery; MVGs (muscle‐in‐vein grafts): *n* = 14 paws at 4 and 8 weeks postsurgery, one animal (two paws) had to be excluded due to missing participation during the training phase; ANG and MVG:* n* = 8 paws at 12 weeks postsurgery). Two‐way ANOVA followed by Tukey's multiple comparison was applied to examine significant differences (***p < .001 vs. 4 weeks postsurgery). Values are displayed as median ± range and given as percentages related to the previously set individual healthy state reference mean values resulting in a healthy state baseline at 100%

During presurgical training, animals were able to reach the highest five steps of the staircases and therefore to eventually retrieve a maximum of 15 pellets (Figure 4a, filled symbols). At the first day of training 4.1 ± 0.9 pellets were retrieved with the right paw and 2.8 ± 0.6 pellets with left paw. At the last day of training, the 15 participating animals had constantly improved to retrieve 10.9 ± 0.9 pellets with their right paw and 7.5 ± 0.8 pellets with their left paw. Twelve of 16 animals retained their initial right paw preference, but the performance did not indicate at any time that the preferred side was recovering earlier than the nonpreferred side. Postsurgical results are displayed as percentages from initial individual reference values (100%) to still exclude any paw preference influences on the final outcome (Figure 4b).

At 4 weeks postsurgery, more than half of the ANG‐reconstructed paws participated in the staircase test (success rates 0.0% to 125.0%, median 21.2% of the maximum pellets retrieved in healthy state). Participation was quite poor in the MVG‐reconstructed animals (only one paw with success rate 50%).

After 8 weeks, both groups showed a significantly increased performance with median success rates close to healthy levels (ANG: 104.8%, MVG: 82.7%). At this time, only three paws of the MVG group were not able to participate.

Until 12 weeks postsurgery, median success rates were constant (ANG: 109.4%, MVG: 89.1%) with still two paws of the MVG group not showing recovery of forelimb reaching skills. Interestingly, several animals were able to retrieve up to the double amount of pellets at this time compared to healthy state. Analyzing the individual performances in more detail, we confirmed that although the animals’ body size and weight increased during the observation period, none of the animals was able to retrieve any pellets from steps 6 and 7 at any time. The detected increase in the overall performance therefore displays an ongoing process of learning (Figure 4b).

### 3.5 Noninvasive electrophysiological recordings

**Figure 5:**
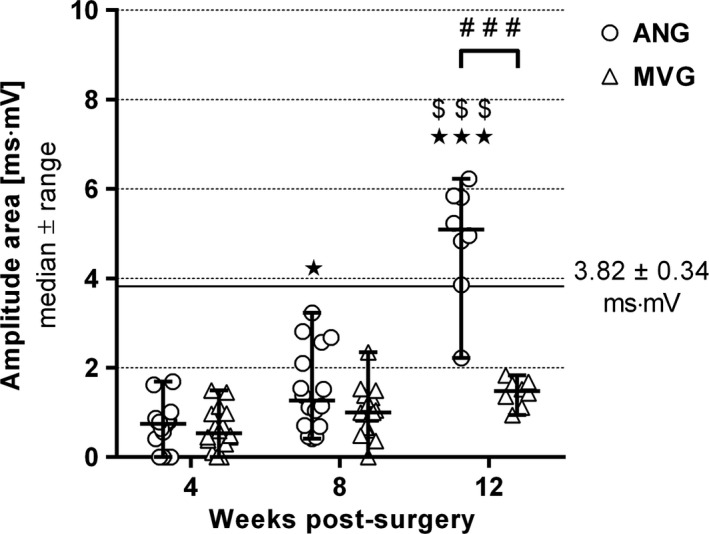
Quantitative results of the electrodiagnostic recordings from the thenar muscle depicting motor recovery over 12 weeks postsurgery. Evoked compound muscle action potentials (CMAPs) were recorded to evaluate an amplitude area over time as common indicator related to motor recovery. While nerve reconstruction with autologous nerve grafts resulted in a significant increase, reconstruction with muscle‐in‐vein grafts led to no significant improvement (*n* = 16 paws evaluated per group at 4 and 8 weeks postsurgery; *n* = 8 paws evaluated at 12 weeks postsurgery). The horizontal continuous line indicates the healthy nerve reference mean value recorded presurgically from *n* = 16 animals. Two‐way ANOVA followed by Tukey's multiple comparison was applied to examine significant differences (*p < .05, ***p < .001 vs. 4 weeks postsurgery; ^$$$^ *p* < .001 vs. 8 weeks postsurgery; ^###^ *p* < .001 as linked). Values are given as median ± range

Following electrodiagnostic recordings, the CMAP amplitude area that correlates in general with the number of regrown axons (irrespective of their diameter or degree of myelination) was evaluated to conclude on the degree of motor recovery (Figure 5).

At 4 weeks postsurgery, evaluation in both groups resulted in approximately the same evoked CMAP amplitude areas ranging at one fifth of initial healthy reference values (horizontal line at 3.82 ± 0.34 ms·mV, Figure 5). Until 8 weeks postsurgery, values in both groups slightly increased with only one MVG‐reconstructed nerve remaining unresponsive. Following removal of four animals per group after 8 weeks, a significant difference between both groups was found at 12 weeks postsurgery. The median amplitude area of the ANG group exceeded the healthy state baseline up to 1.3 times, while the MVG group value remained below 50% of it.

### 3.6 Macroscopic evaluation at the time point of tissue harvest

**Figure 6:**
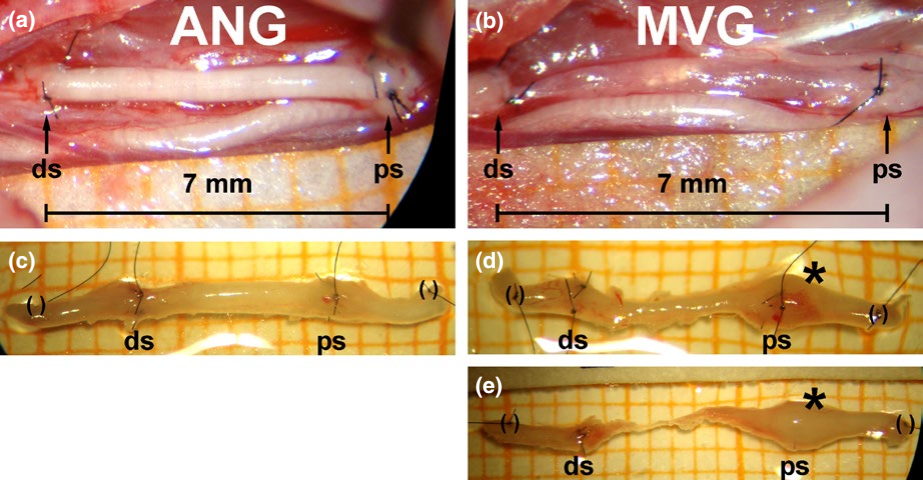
Macroscopic appearance of the sutured grafts during reconstruction surgery (a, b) and of the regenerated tissue upon tissue harvest at 12 weeks postsurgery (c–e). To bridge the previously transected median nerves, either a autologous nerve graft (ANG) (a) or a muscle‐in‐vein graft (MVG) (b) was inserted in a length of 7 mm and subsequently sutured with three stitches at each nerve end (ds = distal suture, ps = proximal suture). Scale paper (millimeter scale) at the bottom of the photomicrographs indicates the distance between the sutures. (c) ANGs showed an unaltered macroscopical appearance upon tissue harvest at 12 weeks postsurgery while (d, e) obvious neuroma formation was found at the proximal suture sites of all MVGs 12 weeks postsurgery (indicated by *; () encircle sutures that were added posttissue harvest to serve as identification marks for immunohistology). While ANGs and MVGs with a good regenerative outcome kept their original length of 7 mm (c, d), MVGs that were stretched up to 10 mm in length (E) led to incomplete functional recovery, although upon following nerve morphometrical evaluation, single axons were found in the distal nerve segments which qualified to induce evocable compound muscle action potentials in electrodiagnostic measurements. Explanted specimens have been placed on scale paper (millimeter scale) in order to indicate the dimensions

Both nerve graft types used differed in their appearance when freshly prepared during surgery, ANGs stayed opaque (Figure 6a), while MVGs appeared to be translucent (Figure 6b). Upon tissue harvest (Figure 6c–e), MVGs adapted to ANGs in their appearance. While macroscopic appearance of ANGs was unaltered (Figure 6c), a more prominent neuroma‐like formation was detectable at the proximal suture sites of all MVGs (indicated by *, Figure 6d,e). All grafts resulting in functional motor recovery kept their initial length of 7 mm (Figure 6c,d). In very few cases, where MVGs were applied but motor skills not recovered, the grafts have been stretched up to 10 mm in length over time (Figure 6e).

### 3.7 Immunohistological evaluation

**Figure 7:**
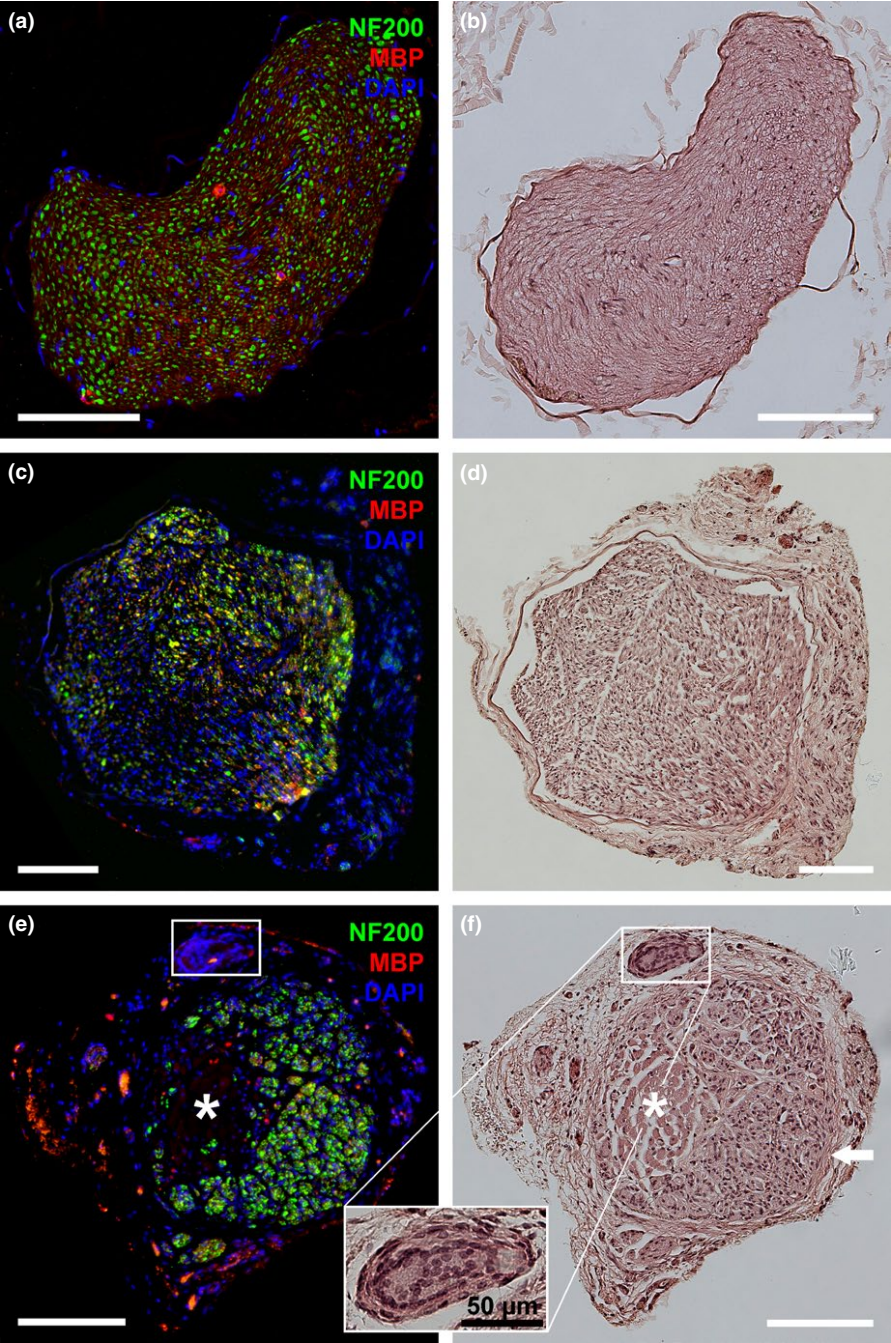
Representative photomicrographs of consecutive cross‐sections through a healthy median nerve (a, b) compared to the regenerated tissue in the middle of either autologous nerve grafts (ANGs) (c, d) or muscle‐in‐vein grafts (MVGs) (e, f) 8 weeks postsurgery. (a, c, e) Immunohistological evaluation displays the immunodetection of healthy and regenerated axons (NF200, green), myelin profiles (MBP, red), and nuclear staining (DAPI, blue). (b, d, f) HE staining allows the discrimination of different tissue types. Healthy nerves revealed dense nerve tissue surrounded only by the epineurium (a, b). ANGs showed a less compact nerve tissue surrounded by a thicker layer of loose connective tissue that had formed around the epineurial layer (c, d). In MVGs, nerve tissue had mainly formed inside the vein walls (indicated by the white arrow) while the regrown nerve tissue has not completely replaced the muscle tissue (indicated by the *) until 8 weeks postsurgery (e, f). Eventually huge cell accumulations were found on the outsides of the vein walls (see box for magnification). (e) In the outer area of the section immunostaining gave some unspecific signals. White scale bars display 150 μm

Cross‐sections of healthy nerves revealed large and evenly distributed NF200‐immunopositive nerve fibers, of which the large diameter ones were surrounded by MBP‐immunopositive myelin profiles (Figure 7a), resulting in a very dense nerve tissue solely surrounded by the epineurium (Figure 7b).

At 8 weeks postsurgery, reconstruction with ANGs (Figure 7c) and MVGs (Figure 7e) led to regeneration of much smaller NF200‐immunopositive spots, irregularly scattered across the whole cross‐section and eventually surrounded by thin MBP‐immunopositive profiles. In contrast to healthy nerve samples, nerve tissue regenerated through ANGs was less compact and encapsulated by an additional thicker layer of loose connective tissue (Figure 7d). HE staining of MVG cross‐sections (Figure 7f) displayed remaining muscle (indicated by *) and vein tissue (indicated by the white arrow). Additionally, few huge cell accumulations (Figure 7f, indicated by the rectangle) were detected on the outside of the vein walls in the surrounding layer of connective tissue in three of seven analyzed MVG grafts.

At 12 weeks postsurgery, ANGs displayed similar tissue properties as before, the residual muscle tissue within MVGs was completely replaced by nerve tissue and cell accumulations were only detected in two of six analyzed MVG grafts.

### 3.8 Histomorphometry

**Figure 8:**
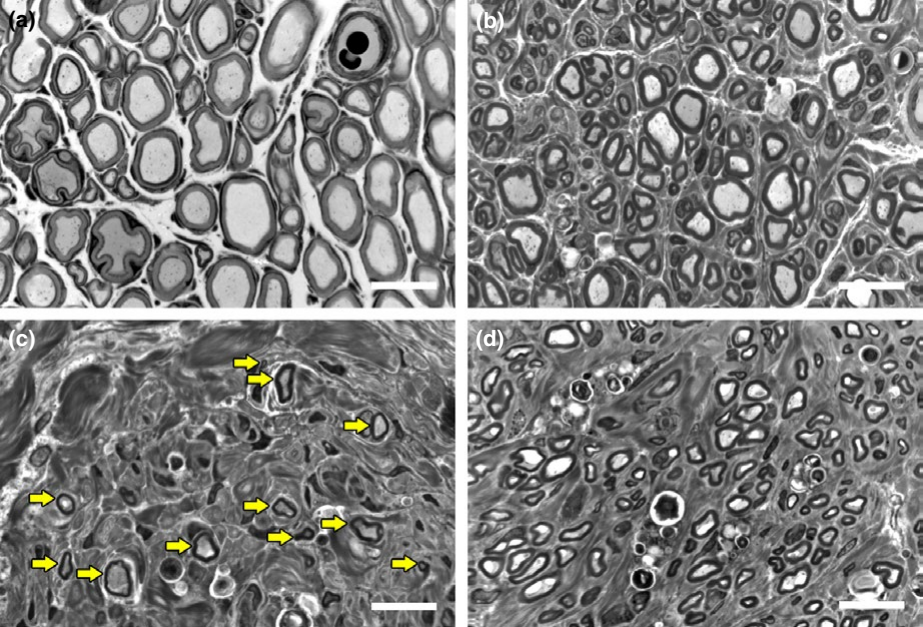
Representative high‐resolution pictures of toluidine blue‐stained semithin cross‐sections showing a healthy distal nerve segment (a) compared to distal nerve segments of reconstructed median nerves (b–d) 12 weeks postsurgery. Images indicate different nerve fiber densities following the application of either autologous nerve grafts (b) or muscle‐in‐vein grafts (MVGs) (c, d) compared to healthy nerve samples (a). Whereas all shown samples led to an evocable compound muscle action potential recorded from the thenar muscle, MVGs with a low nerve fiber density (c; yellow arrows highlight single myelinated axons) did not result in successful participation of the affected forelimbs in the behavioral tests until 12 weeks postsurgery. White scale bars display 15 μm

Semithin cross‐sections of healthy nerve samples (Figure 8a) displayed large myelinated axons, while segments harvested distal to the grafts of ANG‐ (Figure 8b) and MVG‐ (Figure 8c,d) reconstructed nerves displayed myelinated axons smaller in diameter and spaciously distributed. Whereas all cross‐sections of ANG‐reconstructed nerves displayed nearly equal nerve fiber distributions and high nerve fiber densities (Figure 8b), MVGs induced a less dense fiber regeneration ranging from very low (Figure 8c) to higher densities (Figure 8d).

**Figure 9:**
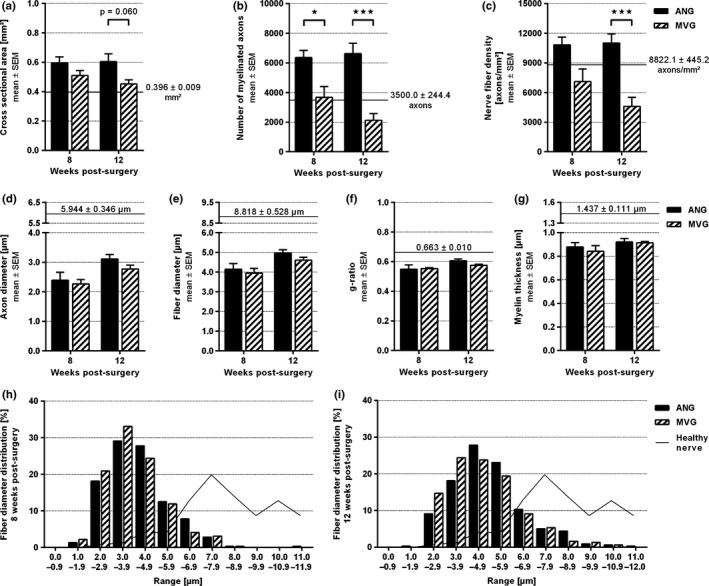
Quantitative results of the nerve morphometrical analysis of healthy nerve segments compared to distal nerve segments of reconstructed median nerves at 8 and 12 weeks postsurgery. Horizontal continuous lines indicate corresponding healthy nerve mean values (*n* = 3). (a–c) Histograms representing the cross‐sectional area of the whole distal nerve segment (a), the total number of myelinated axons (b), and the resulting nerve fiber density (c) (*n* = 8). (d–g) Histograms depicting nerve regeneration‐related size parameters: axon diameter (d), fiber diameter (e), g‐ratio (f), and myelin thickness (g) (*n* = 4). Two‐way ANOVA followed by Tukey's multiple comparison was applied to examine significant differences (*p < .05, ***p < .001 as linked). Values are given as mean ± SEM. (h–j) Histograms illustrating diameter frequency distributions of myelinated nerve fibers 8 weeks (H) and 12 weeks (j) postsurgery obtained by pooling all measured values (*n* = 320 analyzed axons). The fiber diameter distribution of healthy nerve samples is given as a curve in the background (*n* = 240 analyzed axons). Values are expressed as mean percentages

Quantitative evaluation revealed similar cross‐sectional areas in both experimental groups (Figure 9a), although bigger than in healthy nerves (mean value indicated by continuous horizontal line, Figure 9a). Significant differences between the ANG and MVG group were found in the total number of myelinated axons (Figure 9b) resulting in significantly different nerve fiber densities at 12 weeks postsurgery (Figure 9c). Values of total axon count and nerve fiber density derived from healthy nerve samples were lower in comparison to ANG samples or similar/higher compared to MVG samples (Figure 9b,c). Axon and fiber diameter, g‐ratio, and myelin thickness, did not display significant differences between the experimental groups, and did also not reach healthy nerve values (Figure 9d–g). At 8 weeks postsurgery, fiber diameter distributions of both groups were almost similar (ANG/MVG: range 1.0–8.9 μm, peak 3.0–3.9 μm) but displayed a left shift and narrowed range compared to the flattened and wider distribution seen in healthy nerves (range 2.0–17.9 μm, peak 7.0–7.9 μm, mean values indicated by continuous black line, Figure 9h). Until 12 weeks postsurgery, the left shift was slightly corrected towards the healthy nerve fiber diameter distribution (ANG: range 1.0–11.9 μm, peak 4.0–4.9 μm; MVG: range 2.0–10.9 μm, peak 3.0–3.9 μm, Figure 9j).

## 4 DISCUSSION

Both forelimb reaching and grasping tests applied revealed a uniform pattern of recurring motor functions as expectable based on previous reports (Galtrey & Fawcett, 2007). In general, an earlier onset of functional recovery was found in the ANG group leading to full recovery. In contrast, the MVG‐reconstructed animals displayed a delayed and incomplete return of motor skills.

The newly established video‐based scoring system applied with the grasping test provided very detailed and easy‐to‐evaluate information on the onset and progression of individual paw usage abilities of each animal (reflex‐induced fine motor skills). Instead of measuring the grip force of unilateral treated rats (Papalia et al., 2003), our approach (i) reduced stress to the animals eventually induced by means of paw fixation of the uninjured side during the testing procedure (Bertelli & Mira, 1995; Wang, Spinner, et al., 2008) and (ii) minimized animal numbers by avoiding only unilateral treatment following still bilateral injury (Papalia et al., 2003; Sinis et al., 2005). Furthermore, it was less time consuming compared to formerly reported forelimb motion video analysis (Wang, Spinner, et al., 2008). According to findings of others analyzing the performance of ANGs and MVGs after reconstruction of 10‐mm MN gaps with the grasping test (Geuna et al., 2007), we confirmed that ANG reconstruction is more supportive than MVG reconstruction for functional recovery. Little undulation in the degree of motor function displayed in our grasping test results (e.g., ANG group between 9 and 10 weeks postsurgery; MVG group between 10 and 11 weeks postsurgery) very likely relate to a phenomenon also described before (Bertelli & Mira, 1995), the reduction in motivation to participate in the test over time. Nevertheless, only frequent testing at least once a week as in our study allows precise definition of the onset of motor skills recovery.

The additional implementation of the staircase test allowed very sensitive evaluation of motor recovery (Montoya et al., 1991) by means of motivation‐induced reaching behavior but it needs an appropriate training phase (Galtrey & Fawcett, 2007; Nikkhah, Rosenthal, Hedrich, & Samii, 1998; Pagnussat Ade et al., 2009). The test may be inflicted with reduced willingness of certain rat strains to participate in it and their improvement in pellet retrieval during the training phase (Galtrey & Fawcett, 2007; Nikkhah et al., 1998). Especially Lewis rats have been reported to demonstrate quite poor performances during training (Nikkhah et al., 1998), but also later on (Galtrey & Fawcett, 2007). In our study, however, only one of 16 Lewis rats did not participate in the training and had to be excluded from further testing in the staircase. In accordance to another report on paw preferences (Pagnussat Ade et al., 2009) in this test, a significant preference for the right paw was detected during training in 12 of 16 animals while none of our animals preferred the left paw. Due to an ongoing learning process while the individual success rates were recorded postsurgically, some animals were increasing their success rates with functional recovery above presurgical values.

Comparing both motor skills tests in more detail, we found very little differences as for example seen in the MVG group at 4 and 12 weeks postsurgery. After 4 weeks postsurgery, no finger flexion at all could be observed in the MVG group during the grasping test (reflex‐induced grasping) while in the staircase test already one single paw was able to reach 50% of its presurgical performance (motivation‐induced forelimb reaching). The same phenomenon was seen at 12 weeks postsurgery, when in the grasping test three paws showed no finger flexion while in the staircase test only two paws were not able to participate. These findings perfectly reflect why both tests should be used in parallel as already assumed by others before because both are affected by possible misdirection of axonal regeneration following complete transection injuries (neurotmesis) (Galtrey & Fawcett, 2007). Fine and gross motor skills as combinatorial evaluated in the grasping and staircase test require accurate motor reinnervation as well as a considerable amount of reinnervation. Grip force is commonly evaluated in the grasping test and is strongly dependent on most accurate and complete target muscle reinnervation. Therefore, especially recovery of grip force is the last section of the peripheral nerve regeneration process to be acquired.

Moreover, purely quantitative information on the degree of motor reinnervation could be obtained by evaluating CMAP amplitude areas. Only few previous studies included recordings of evoked CMAPs in the respective muscles when applying the rat MN model. In these studies, measurements were performed either repeatedly in a noninvasive manner (Wang, Sorenson, et al., 2008) or once invasively at the end of the studies (Sinis et al., 2005; Werdin et al., 2009). Others concluded that invasive endpoint measurements of evoked CMAPs in correlation with histomorphometrical results are a more sensitive way to evaluate axonal regeneration than performing functional tests, especially after functional recovery was already completed (Manoli, Werdin, et al., 2014). However, our results point to the benefit from monitoring the early onset and progression of motor recovery by noninvasive recordings at different desired time points. This clearly reveals differences in the performance of nerve graft types in supporting earlier return of function and earlier completion of recovery, representing parameters highly valuable to patients. With increasing availability of alternative tissue‐engineered nerve grafts with eventually only slight refinements distinguishing one from the other, more comprehensive preclinical studies are warranted. Furthermore, we found a time shift in the CMAP recordings when comparing the derived results to those results derived from the behavioral tests. The electrodiagnostic findings displayed an earlier onset of motor recovery than assumed from the two other tests. Already at 4 weeks postsurgery, more than 80% of the reconstructed nerves transmitted an evocable CMAP independent of the graft type used. At this time point, however, only a few animals of the ANG group were able to catch a relevant number of pellets in the staircase test while still not demonstrating a remarkable grip force in the grasping test. Similar findings were achieved at 12 weeks postsurgery, when two MVG‐reconstructed nerves have not still led to functional recovery, but evocable CMAPs were found in all animals. Quantitative analysis of the CMAP signals was, however, demonstrating significant differences between both groups. The CMAP amplitude area demonstrates the same progression of motor recovery in our study, but we found larger CMAP areas in the ANG group compared to the healthy state recordings. As the CMAP area correlates with the number of available functioning axons (Cuddon, 2002), a higher number of regrown nerve fibers can be assumed in the ANG‐reconstructed nerves compared to uninjured rat MNs. Nevertheless, the exclusive detection of CMAPs does not allow a reliable conclusion on the degree of recovered motor skills as misdirected axonal regeneration following neurotmesis strongly affects the same.

However, immunohistological and histomorphometrical evaluation of the harvested nerve tissue within this study confirmed axonal regeneration in all grafted nerves as formerly expected from the electrodiagnostic results. Immunohistological evaluation gives additional insight into the integration of the applied grafts with the host tissue. At 8 weeks postsurgery, for example, we still detected muscle and vein tissue in all MVGs and few huge cell accumulations in the outer connective tissue close to the vein walls in a limited number of MVGs. These cell formations are similar in their appearance to macrophage giant cells that one eventually observes as an immune response to implanted materials (Haastert‐Talini et al., 2013). Therefore, we conclude that these cells might be involved in the remodeling process of ectopic autologous vein tissue into nerve fascicle ensheating tissue. At 12 weeks postsurgery, all muscle tissue was completely replaced by regenerating axons as already reported by others (Brunelli, Battiston, Vigasio, Brunelli, & Marocolo, 1993). Histomorphometrical analysis further elucidates quantitative parameters of axonal regeneration in distal nerve segments. In accordance to previous reports (Ronchi et al., 2014; Sinis et al., 2005), ANG‐reconstructed nerves display much higher numbers of myelinated axons than healthy or MVG‐grafted nerves. Most importantly, this confirms our results of the electrodiagnostic measurements with increased CMAP areas in the ANG group indicating a higher amount of regrown axons. Our results of the size parameter evaluation are in correspondence to the known fact that nerve fibers cannot return to their presurgical size even after very long recovery times (Muratori et al., 2012).

**Figure 10:**
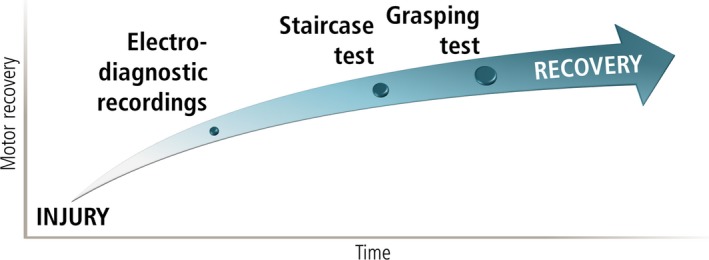
Schematic drawing of the progress in motor recovery over time as detectable with the three different periodically applied noninvasive functional evaluation methods

Finally, comparing the two nerve graft types used in this study, we have to assume that in our hands, MVGs led to a delayed onset of functional recovery and to an overall reduced rate of recovery in comparison to the gold standard ANG repair. Some earlier experimental (Brunelli et al., 1993) and recent clinical work (Manoli, Schulz, et al., 2014) did, however, not report any inferiority of MVGs in comparison to ANGs especially when shorter nerve defects have been bridged. Despite the fact, however, that MVGs also resulted in considerable recovery of fine and gross motor skills in our study, the multimodal analysis of functional recovery performed was comprehensive and therefore eventually more sensitive to otherwise undetectable variations between the graft types. Overall, rat MN reconstruction with ANGs resulted in earlier and more complete axonal and functional recovery than reconstruction of the nerve lesions using MVGs. As illustrated in Figure 10, the advancement of the rat MN injury and reconstruction model by combining three different periodically applied noninvasive functional evaluation methods revealed distinct levels of regeneration. Final histomorphometrical analysis underscored the previous functional findings.

Therefore, the specific experimental design allows (i) very precise observation of the onset, progression, and completeness of functional recovery in bilaterally reconstructed animals, (ii) reduction in animal numbers due to video analysis, and (iii) monitoring of the speed and amount of axonal regeneration as well as the accuracy of motor reinnervation in dependency of the graft type used.

Especially the advantage of being able to distinguish between graft types that induce earlier recovery of motor skills while eventually resulting in the same degree of motor function after regeneration is completed provides a very useful tool in translational research. The earlier a patient demonstrates recovery of motor function the earlier his/her quality of life is increased as is the socioeconomic burden reduced which is commonly raised by severe PNIs. We therefore strongly recommend this valuable advanced rat MN injury and repair model to study novel tissue‐engineered nerve graft approaches in preclinical translational studies.

## CONFLICT OF INTEREST

None declared.
